# Supplementary material for: Physical Activity during the COVID-19 Pandemic in the UK: A Qualitative Analysis of Free-Text Survey Data
Source: Int J Environ Res Public Health. 2022 Nov 10;19(22):14784. doi: 10.3390/ijerph192214784 (PMC9690793; doi:10.3390/ijerph192214784)
Supplement: Supplementary file 1 [file ijerph-19-14784-s001.zip › ijerph-1943040-supplementary.pdf]

International Journal of
Environmental Research and Public Health

MDPI

Supplementary information

Table S1. Timeline of COVID-19 restrictions in England 2020 [50], [51].

Date	COVID-19 restriction
Mid-March to mid-May (Full lockdown	National restrictions (lockdown 1). Sports facilities closed, restrictions applied to all indoor activity and organised outdoor activity. Single daily allowance of activity allowed.
Mid-May	Unlimited outdoor exercise is permitted. Meeting one person outside your household outdoors allowed. Activity choice extended to include water sports
June	Schools reopened (year 1 to year 6). Groups of 6 people allowed to meet outdoors (rule of 6). Indoor gatherings of two people from different households permitted. Outdoor training sessions allowed.
Early July	Local Lockdown regulations introduced and used
Mid-July	Playgrounds & outdoor pools reopened. Organised sports restarted
End- July	Gyms, indoor pools & leisure centres reopened
Sept	Schools reopened to all age groups
Mid-Sept to Oct*	Restrictions reintroduced for indoor team sports. Reintroduction of maximum of 6 people allowed to meet outdoors (rule of 6)
November	National restrictions (lockdown 2). Restrictions applied to all indoor activity and organised outdoor activity
December	New tiered system of restrictions based on location

*Wales had an additional 'fire-break' lockdown 23/10/20 and 09/11/20.

Table S2. Physical activity words.

Letter	Physical activity related word
**A**	activities act4ivity activates active active_ actively actives activiites activites activities activities
**B**	badminton ball ballet ballets ballroom balls basketball bicycle bicycles bike bikes biking bikr boat boating bodybuilder bodyweight bowling bowls breathlessness brisk briskly
**C**	canoe canoeing cardiac cardic cardio chi class classes club clubs club's coach coached coaches coaching competing competition competitions competitive competitively competitiveness couch2 courts cricket crossfit crosstrainer curcuit curling cycle cycled cycles cycling cyclist cyclists
**D**	dance dancefit danceing dancer dancers dances dancesing dancing diving dogwalkers dogwalking
**E**	ecercise endurance energetic energy excercies excercise excercises excercising

	excerise excersise excersising excersize excise execise execising execrcise exe-crise exercis exercise exercise.eating exercised exerciser exercises exercising exericse exerrcise exersice exersise exersize exrecise
**F**	fatter fit fitbits fitbut fitness fitter fittest football footballer footballers footie footing
**G**	gardened gardener gardening garmin golf golfer golfers golfing gym gymn gymnastics gyms gym's
**H/I**	hike hiked hikes hiking horse horsemen horseracing horses inactive inactivity
**J**	joe joes jog jogged jogger joggers jogging juggle juggled juggling
**K/L**	kayak kayaking kettlebells kick kickboxing kicked kicking lido
**M/O**	marathon marathons member members member's membership memberships mileage miles mountain mountaineering mountains outdoorsy
**P**	padding paddle paddling padel parkrun parkruns parkrun's phyical phyicsal physcial physical physically pilates play players playground playgrounds playing plod plodded plodding plods ponies pony pool pools powerlifting
**R**	race races race's racing racket racketball ramble rambler ramblers rambling riding rugby runner runners running runs
**S**	scuba sculling shoot shooting shoots skate skated skating ski skiing skip skipping skips snowboarding soccer sport sportcentre sporting sports sportsmen sporty sprint sprints strava stretch stretched stretches stretching stroll strolls sweat sweating sweats sweaty swim swim2 swimmer swimmers swimmin swimming swimminh swimmng swims
**T**	train trainer trainers training trampoline trampolining
**U/V**	ultra ultras vegetate vegetating vegging
**W/Y/Z**	walker walkers walking walking.exercising walks watersports weight weightlifting weights workout workouts yoga yoga's zumba
**Numbers**	10,000 10000 _outdoor 10,000s 10000s 10k 10km 11k 12k 1k 1km 20k 20km 21k 2kms 2miles 5km 5ks 5miles

Table S3. Example Excel coding.

First order	PA	Change	Barrier (1)	Barrier (2)	Facilitator (1)	Facilitator (2)	Impact	ID	Quote
1	gardening	increase			garden			1234	*Lots of gardening has kept me going*
1	exercise	not clear			nature	outdoors	mental	1345	*Exercise- getting out into nature has kept me sane*
1	walk	same			dog	social contact		1456	*Having a dog is a fantastic outlet for me. From chatting to her and walking every day to meeting other dog walkers whilst out walking and I've been able to have socially distanced chat with them*
1	exercise	increase			weather			1567	*Trying to do more exercise but only achieve when weather was good*
1	exercise	increase			time			1678	*Working from home means I don't need to spend time travelling to work. I can use this extra time for other activities such as exercise*
2	walk	decrease	active commute				physical	1789	*Not walking to work every day has had a big impact on my physical health – I have become a couch potato*

2	exercise	de-crease	time					2234	*My exercise is really low as I can't get out to do things with children at home*
2	gym	de-crease	closure				mental	2245	*Not being able to go to the gym during lockdowns had an impact on mental health*
2	walk	de-crease	gov restriction				well-being	2256	*I have had fewer opportunities to go walking with friends in the mountains which I normally do each week. This has led to reduced fitness and loneliness*
2	classes	de-crease	covid	safety			well-being	2267	*I have stopped going to my exercise classes and I miss that, but when they did start up again, I didn't want to go back due to the worry of catching the virus*
2	walk	de-crease	weather	safety				2278	*I don't like going out on my own for walks and now its darker much earlier I'm not walking on an evening with my husband so rarely leave the house*
1	exercise	in-crease			health		well-being	2289	*I have made my health a priority now so that if I did catch Covid I am in better shape to tackle it. I now exercise far more and am losing weight, this has had a positive impact on how I feel about myself*
1	yo-ga/pilat es	in-crease			tech	time		3345	*Positive impact has been an increase in exercise as zoom has enabled me to prac-tice yoga and Pilates at times that suit me better from my spare room*
3	yo-ga/pilat es	not clear			tech			3356	*I do yoga online but miss being able to do it in a studio with other people*
1	exercise	in-creased			health		mental	3367	*I started exercising every day during the first lockdown and although I now only do 3-4 days a week, I think it helped my mental health*
